# The development of complex tooth shape in reptiles

**DOI:** 10.3389/fphys.2014.00074

**Published:** 2014-02-25

**Authors:** Oldrich Zahradnicek, Marcela Buchtova, Hana Dosedelova, Abigail S. Tucker

**Affiliations:** ^1^Department of Teratology, Institute of Experimental Medicine, v.v.i., Academy of Sciences of the Czech RepublicPrague, Czech Republic; ^2^Laboratory of Animal Embryology, Institute of Animal Physiology and Genetics, v.v.i., Academy of Sciences of the Czech RepublicBrno, Czech Republic; ^3^Department of Anatomy, Histology and Embryology, Faculty of Veterinary Medicine, University of Veterinary and Pharmaceutical SciencesBrno, Czech Republic; ^4^Department of Craniofacial Development and Stem Cell Biology, and Department of Orthodontics, King's College London, Guy's HospitalLondon, UK

**Keywords:** odontogenesis, reptile, cusp, crest, crown

## Abstract

Reptiles have a diverse array of tooth shapes, from simple unicuspid to complex multicuspid teeth, reflecting functional adaptation to a variety of diets and eating styles. In addition to cusps, often complex longitudinal labial and lingual enamel crests are widespread and contribute to the final shape of reptile teeth. The simplest shaped unicuspid teeth have been found in piscivorous or carnivorous ancestors of recent diapsid reptiles and they are also present in some extant carnivores such as crocodiles and snakes. However, the ancestral tooth shape for squamate reptiles is thought to be bicuspid, indicating an insectivorous diet. The development of bicuspid teeth in lizards has recently been published, indicating that the mechanisms used to create cusps and crests are very distinct from those that shape cusps in mammals. Here, we introduce the large variety of tooth shapes found in lizards and compare the morphology and development of bicuspid, tricuspid, and pentacuspid teeth, with the aim of understanding how such tooth shapes are generated. Next, we discuss whether the processes used to form such morphologies are conserved between divergent lizards and whether the underlying mechanisms share similarities with those of mammals. In particular, we will focus on the complex teeth of the chameleon, gecko, varanus, and anole lizards using SEM and histology to compare the tooth crown morphology and embryonic development.

## Introduction

Teeth come in many shapes and sizes, this variation allowing animals to take advantage of very different food types, and influencing social behavior. The most simple shaped teeth are conical, cylindroconical, flattened or slightly bent, terminating in a single cusp, such as observed in many snakes, monitor lizards and crocodiles for holding onto prey, and transporting it into the esophagus. The most complex teeth, as observed in animals such as the giant panda, are large with multiple cusps for crushing and grinding (Evans et al., 2007; Ungar, 2010). How cusps form during embryonic development in mammals has been carefully followed in species such as the mouse, where a structure known as the enamel knot has been shown to be of central importance (Jernvall et al., 1994). The enamel knot forms within the inner enamel epithelium and it signals to the surrounding tissue. The enamel knot itself does not proliferate, while the surrounding tissue does resulting in the folding of the inner dental epithelium. This folding creates the tooth shape. Once the folds have been created the primary enamel knot undergoes apoptosis (programmed cell death) leading to its silencing as a signaling center (Jernvall et al., 1998). Single cuspid incisors only have one enamel knot, while multicuspid molars have additional secondary enamel knots (SEK) that further fold the inner enamel epithelium and create additional cusps. The SEKs sit at the sites of the future cusps, with the number of SEK correlating with the number of cusps in the final tooth. The position and time of induction of the SEK is also central to the final tooth shape, by controlling the location and pattern of the final cusps in different species (Jernvall et al., 2000; Moustakas et al., 2011; Jernvall and Thesleff, 2012).

Multicuspid teeth, however, are not restricted to the mammals and are found in fish and reptiles (Edmund, 1969; Streelman et al., 2003). Whether the cusps in these teeth are generated by a similar mechanism to that of the mammals is much debated. Evidence has been presented for and against the presence of an enamel knot type structure in these groups. An enamel knot would be characterized by a thickened part of the inner dental epithelium showing lack of proliferation, high apoptosis and the expression of key signaling molecules, such as *Shh* (Sonic hedgehog) and *Fgf4* (Jernvall et al., 1994, 2000; Matalova et al., 2004). A slight thickening of the inner dental epithelium has been reported in the alligator (Westergaard and Ferguson, 1987), and a bulge of the inner dental epithelium associated with apoptotic bodies has been observed in the chameleon, indicating a possible enamel knot (Buchtova et al., 2013). No such bulge has been shown in the unicuspid snake, however, at the cap and bell stage of python tooth development some signaling molecules (*Wnt 6*), and pSMAD activity have a localized expression in the center of the inner enamel epithelium reminiscent of a primary enamel knot (Handrigan and Richman, 2010, 2011; Richman and Handrigan, 2011). On the other hand, many other signaling molecules expressed in the murine primary enamel knot, such as *Edar*, *Shh*, and *Bmp4*, do not share this localization but are more wide spread in the inner enamel epithelium (Handrigan and Richman, 2011). Proliferation is absent from the center of the inner enamel epithelium in snakes, as associated with an enamel knot, however, apoptosis is not observed within the inner enamel epithelium but instead is located in the underlying stellate reticulum layer (Buchtova et al., 2008).

We have investigated this further by studying cusp development in several reptile species, a unicuspid snake (*Python molurus*), a bicuspid gecko (*Paroedura picta*), multicuspid lizards – the chameleon (*Chamaeleo calyptratus*), four anolis (*Anolis barbatus, Anolis porcus, Anolis baracoae, Anolis allisoni)*, and Nile monitor lizard (*Varanus niloticus)*. The unicuspid tooth of the snake allows the animal to hold its large prey while it is ingested whole, the bicuspid tooth of the gecko allows it to bite small prey, such as insects, while the tricuspid teeth of the chameleon and anolis allows them to crush larger insects, small vertebrates, plant matter (in the case of the chameleon), and hard molluscs (in the case of the False Cuban anole) (Herrel and Holanova, 2008). In each case, we have looked at the morphology of the inner enamel epithelium as the crown developed. We have found two distinct structures developing on the crown – enamel crests and dental cusps. Their appearance and morphology is species specific and they appear to develop by very different but universal mechanisms.

## Materials and methods

### Animals

Reptilian specimens were obtained from private breeders, and the Faculty of Sciences, Charles University in Prague (Czech Republic). Embryos and fetuses were euthanized by MS222 and fixed in 4% PFA. Decapitation was additionally used in the case of fetuses before fixation. Juveniles died naturally and were frozen or fixed in 70% ethanol by breeders before further analysis. All procedures were conducted following a protocol approved by the Ethical committee IAPG CAS v.v.i.

**Table:** 

**English name**	**Latin name**	**Family**	**Number of tooth generations**	**Tooth shape**
Indian python	*Python molurus*	Pythonidae	Multiple	Simple conical
Nile monitor	*Varanus niloticus*	Varanidae	Multiple	Cusps
Madagascar ground gecko (Ocelot gecko)	*Paroedura picta*	Gekkonidae	Multiple	Crests
Veiled chameleon	*Chamaeleo calyptratus*	Chamaeleonidae	One	Cusps and crests
Western bearded anole	*Anolis barbatus*	Dactyloidae	Multiple	Cusps and crests
Orient bearded anole	*Anolis porcus*	Dactyloidae	Multiple	Cusps and crests
Baracoa cuban anole	*Anolis baracoae*	Dactyloidae	Multiple	Cusps and crests
Allison's anole	*Anolis allisoni*	Dactyloidae	Multiple	Cusps and crests

### Sem

Dead juvenile animals were collected and fixed in 70% ethanol before scanning electron microscopical analysis of the surface structure. Lower jaws were carefully cleared of soft tissue by using forceps and bleaching solution. The samples were dehydrated in a graded ethanol series and placed in 100% ethanol. Just before scanning, they were air-dried, glued onto an aluminium support and coated with a thin layer of gold and analyzed in a JEOL SEM 6380 LV.

Following recent papers, we use anterior and posterior when discussing tooth position in relation to the jaw and mesial or distal and labial or lingual for individual sides of a tooth.

### Histological analysis

Embryos and fetuses were embedded in paraffin and sectioned for 5–10 μm serial tissue sections and stained with Haematoxylin-Eosin or Haematoxylin-Eosin-Alcian blue.

## Results

### Variation in reptilian tooth shape

**Figure 1:**
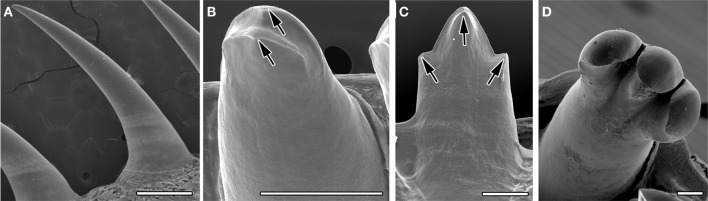
**Tooth crown variety in reptiles**. SEMs of adult teeth. **(A)** Unicuspid snake, *Python molurus*, **(B)** two crests in gecko, *Paroedura picta*, arrowed. **(C)** Tricuspid anole, *Anolis allisoni*. Cusps arrowed. **(D)** Tricuspid Monitor lizard, *Varanus niloticus*. Scale bar = 500 μm **(A)** and 100 μm **(B–D)**.

Eight species of reptile (four anoles, chameleon, gecko, python, and varanus) were analyzed to investigate tooth shape using scanning electron microscopy (SEM). The shape of the crowns ranged from the simple unicuspid teeth of the python (Figure 1A), to the highly complex teeth of the Nile monitor lizard, *Varanus niloticus* (Figure 1D). In some species such as the gecko, two crests were apparent at the tip of the tooth crown, bifurcating the crown in the lingual-lingual orientation (Figure 1B). Anoles exhibited tricuspid teeth with two additional lateral cusps on either side of a central cusp, on the posterior teeth. The central cone of these multicuspid teeth was split into labial and lingual crests, in a manner similar to the gecko (Figure 1C). The dentition of the juvenile of *Varanus niloticus* was composed of triconodont teeth with large lateral cusps (Figure 1D) prominent particularly in the upper jaw attaching to the premaxillary bone. This large variation in the crowns of reptilian teeth appears driven by a wide variety of diets.

**Figure 2:**
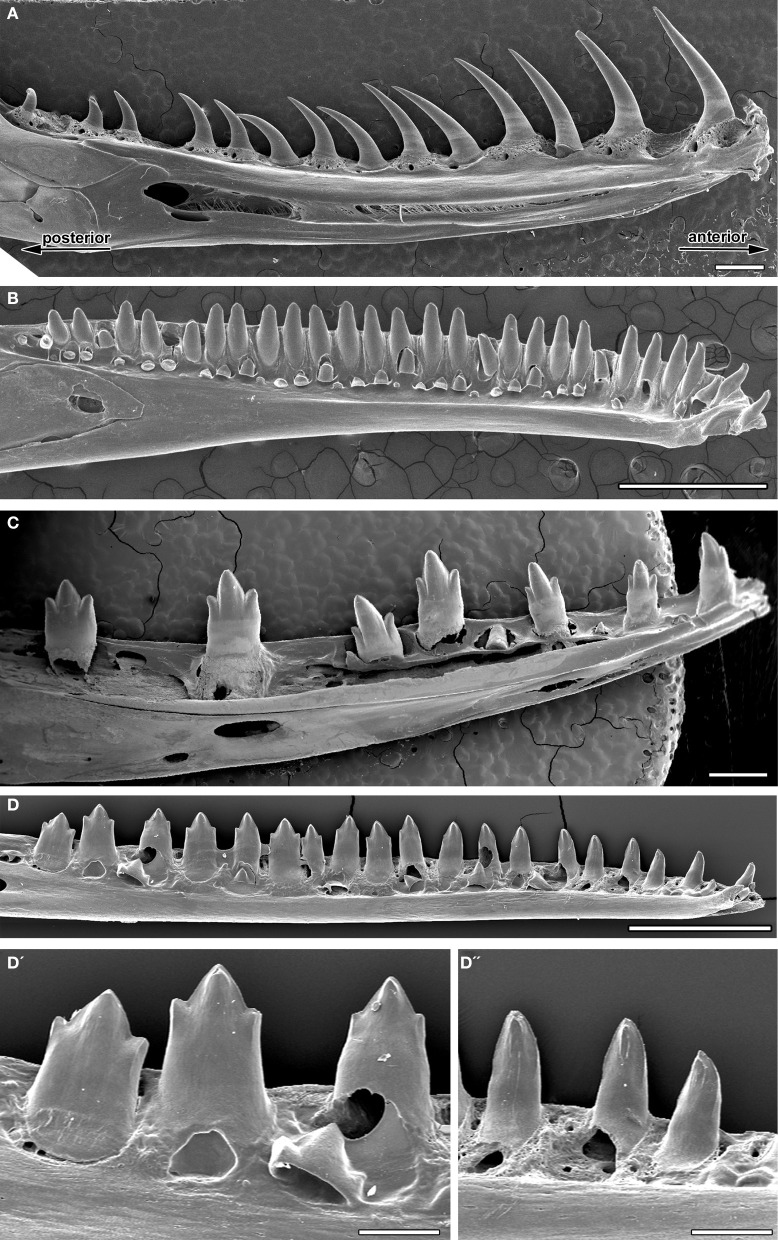
**Tooth shape changes within the jaw (heterodont/homodont)**. SEMs of adult lower jaws. **(A)** Homodont unicuspid snake, *Python molurus*, **(B)** homodont gecko, *Paroedura picta*, **(C)** homodont tricuspid Monitor lizard, *Varanus niloticus*, **(D)** heterodont anole, *Anolis allisoni*. **(D′)** Tricuspid teeth of the posterior jaw at the back of the mouth. **(D″)** Unicuspid teeth of the anterior jaw at the front of the mouth. Scale bar = 1 mm **(A–D)** and 200 μm **(D′,D″)**.

Within the amniotes, a homodont dentition is usually associated with reptiles. A classic homodont pattern was found in the gecko (Figure 2B) with similar sized bicuspid teeth along the whole jaw. This was also observed in *Varanus niloticus*, where tricuspid teeth were found along the dentary and maxillary bones (Figure 2C). In the python, although a unicuspid tooth shape was universally observed, the size of the teeth varied considerably, with a gradient in size from anterior to posterior (Figure 2A). Interestingly, however, a large variation in tooth shape was found across the jaw, in all the anoles studied (*A. baracoae, A. barbatus, A. porcus, A. allisoni*). In these reptiles, the anterior teeth were unicuspid in shape while the teeth further back in the jaw were tricuspid (Figures 2D,D′,D″ and data not shown).

### Formation of crests

**Figure 3:**
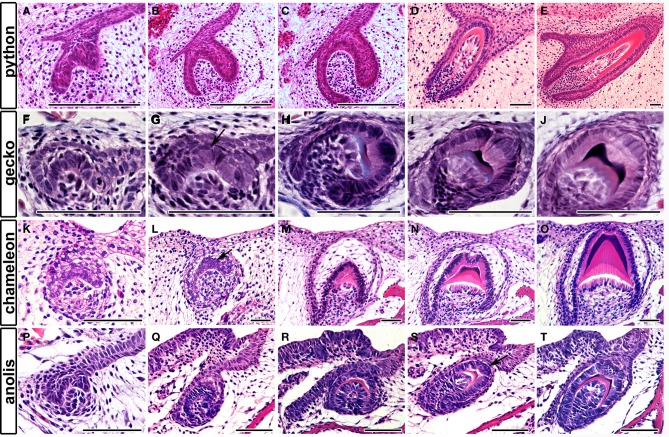
**Tooth crest formation and the epithelial bulge**. Frontal sections through reptilian teeth as they develop from cap to bell stage and final differentiation. **(A–E)** Unicuspid snake, *Python molurus*. No bulge is found in the inner enamel epithelium. **(F–J)** Bicuspid gecko *Paroedura picta*. A bulge is found in the central part of the inner enamel epithelium. See arrow in **(G)**. **(K–O)** Lingual-labial crest formation in the central cusp of the chameleon, *Chamaeleo calyptratus*. **(P–T)** Lingual-labial crest formation in the central cusp of the anole. *Anolis allisoni*. A bulge is found in the inner enamel epithelium in both species. See arrow in **(L,S)**. Scale bar = 50 μm.

The formation of complex teeth, where the crown is divided into labial and lingual crests has been recently followed in two species of gecko, the leopard gecko, *Eublepharis macularius* (Handrigan and Richman, 2011) and the Madagascan ground gecko, *Paroedura picta* (Zahradnicek et al., 2012). In both species, an epithelial bulge was reported during development at the center of the inner enamel epithelium at the cap stage (Figures 3F,G), the crests forming due to asymmetrical deposition of enamel around this region (Figures 3H–J). The changing thickness of enamel therefore creates the crests, which sit on a rounded unicuspid dome of dentin. Unicuspid teeth, such as those in snakes, in contrast do not have a similar epithelial bulge (Figures 3A–E) (Buchtova et al., 2008; Handrigan and Richman, 2011). Similar labial-lingual crests, however, are observed in other unrelated reptile species, such as the chameleon and anole, and therefore we wished to investigate whether these crests are also created by a similar mechanism. The chameleon possesses tricuspid-pentacuspid teeth in the central and posterior part of the jaw, each tooth formed of a central cusp surrounded by accessory cusps formed along the anterior-posterior axes. The central cusp, however, also has crests on the top of its crown, found labial-lingually. The formation of these crests can be followed during development in frontal/transverse section. Similar to the gecko a bulge in the center of the inner dental epithelium was evident at the cap stage (Figures 3K,L). This led to a change in arrangement of the developing ameloblasts at the very tip of the crown, forming a U shaped arrangement as development progressed (Figures 3M,N). As in the gecko the central cusp crests were formed by asymmetrical deposition of enamel by the ameloblasts, sitting on top of a unicuspid dentin (Figure 3O). A similar epithelial bulge and the start of asymmetric enamel deposition was observed in the anole on the central cusp (Figures 3P–T). Formation of crests therefore follows a similar pattern in unrelated reptiles.

### Formation of cusps

**Figure 4:**
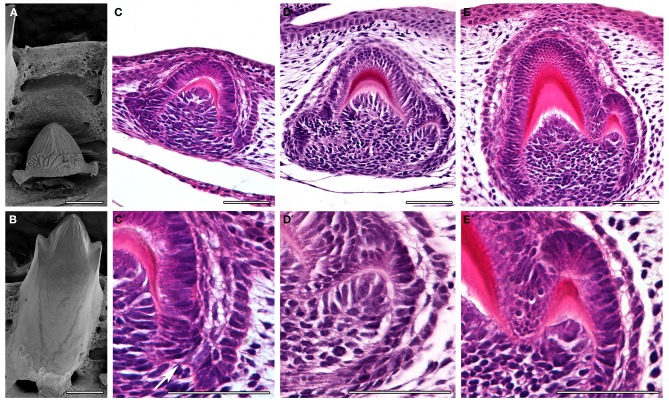
**Tooth cusp development in the anole, *Anolis allisoni*. (A,B)** SEMs of a developing unerupted tooth **(A)** and a functional tooth **(B)**. **(C–E)** Sagittal sections through developing anole teeth at stage 17 and 18. **(C′–E′)** High power views of the images above. The cusps can be seen to form by folding of the inner enamel epithelium. See arrow in **(C′)**. Scale bar = 100 μm **(A,B)** and 50 μm **(C–E, C′–E′)**.

**Figure 5:**
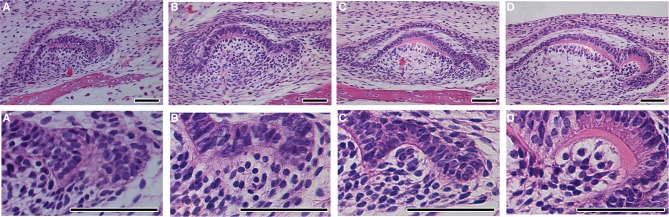
**Tooth cusp development in the chameleon. (A,A′)** Tooth at the cap stage with no deposition of hard tissue. A developing accessory cusp is visible on the RHS. **(B,B′)** The inner enamel epithelium on the RHS starts to fold, associated with a ball of underlying mesenchymal cells. **(C,C′)** Dentine starts to be deposited in the central crown. **(D,D′)** Dentine starts to be deposited in the accessory crown. **(D′)** the dentine of the central crown has yet to merge with that of the accessory crown. Scale bar = 50 μm.

The crests previously described are rather different from the cusps of mammals, which have been shown to be formed by a folding of the inner enamel epithelium prior to the onset of differentiation and deposition of hard tissue. How cusps are formed in tricuspid reptile teeth has been largely ignored. We therefore, followed the development of tricuspid teeth in the anole (Figure 4) and chameleon (Figure 5). In the anole the central cusp formed first followed by the accessory cusps. This pattern is clear when comparing the relative size of the cusps in mineralized developing teeth (Figure 4A), compared to the functional teeth (Figure 4B). Teeth were sectioned sagittally in order to view the microscopic anatomy of the developing cusps. After formation of the central cusp, the epithelium of the cervical loops started to fold at the edges of the tooth to create the accessory cusps (Figure 4C). On folding to form the accessory cusps, the cells of the inner enamel epithelium changed shape, such changes presumably leading to bending of the epithelium (Figure 4D). The central and accessory cusps mineralized from their tips, with the central cusp mineralizing first, the hard tissues ultimately fusing at the base to unite the cusps (Figure 4E). To look at the folding process in more detail, we investigated the formation of the accessory cusps in the chameleon in teeth of slightly different age within the same jaw. As in the anole the central cusp developed first, but in the chameleon the accessory cusps appeared to initiate before mineralization of the central cusp (Figures 5A,B). Again cusp formation was driven by folding of the inner enamel epithelium, these cells appearing to rearrange to create an elongated layer of ameloblasts around the new cusp (Figures 5C,D). Folding of the epithelium appeared to be associated with the formation of a discrete ball of cells in the underlying mesenchyme (Figure 5C′), perhaps indicating the involvement of epithelial and mesenchymal interactions in cusp formation. As in the anole, mineralization initiated at the tips of the cusps with the layers ultimately fusing. This folding of the epithelium, before deposition of hard tissue, to create the tooth shape is reminiscent of that observed in mammals (mouse, vole, shrew, opossum) (Moustakas et al., 2011; Jernvall and Thesleff, 2012).

## Discussion

Our study highlights the fact that reptilian teeth exhibit a large variety of tooth shapes closely associated with diet. The dentition can be heterodont, showing conical teeth in the anterior of the mouth and more complex teeth posteriorly, similar to many mammalian dentitions.

Our analysis of crown crest development indicates that asymmetrical deposition of enamel is a universal mechanism to form such dental ornaments in reptiles, with the creation of an epithelial bulge central to this mechanism. Interestingly, asymmetrical deposition of enamel is also observed in mammals and fish as a way to create more complex tooth shapes. For example, in many rodents the incisors are grooved created by enamel free zones (Ohazama et al., 2010). Mouse and rat molar teeth also have enamel free zones associated with the tips of the cusps, associated with a very thin layer of enamel-like matrix (Sakakura et al., 1989; Yamamoto et al., 1998). The ameloblasts at the enamel free areas have been termed non-formative or functionless ameloblasts (Gaunt, 1956; Cohn, 1957). This region of the inner enamel epithelium expresses *Slit1*, and has been termed a tertiary enamel knots (TEK) (Luukko et al., 2003). The cells in the TEK undergo apoptosis, similar to secondary and primary enamel knots (Hu et al., 2011). It is tempting to speculate that the epithelial bulges observed during reptile crest development might be similar in function to such mammalian TEKs.

In the gecko, expression of *Bmp2* in the enamel bulge has been suggested to control ameloblast differentiation in this region (Handrigan and Richman, 2011) and modulation of BMP activity has been proposed to play a role in creation of enamel free areas in mammals (Ohazama et al., 2010). Therefore, BMPs may play a general role in controlling the differentiation of ameloblasts.

That true cusps in reptiles are formed from folding of the inner enamel epithelium indicates common mechanisms for cusp development between reptiles and mammals. Cusp development, however, also showed some differences. In the anole, the central cusp formed first, followed by the lateral accessory cusps, with the central cusp differentiating ahead of the folding process, in contrast to the mouse where the complete cusp pattern is generated before differentiation starts. In the chameleon, the accessory cusps appeared to initiate before the central cusp underwent differentiation, showing a slightly different relative timing of this process when compared to the anole. In both reptiles, however, the hard tissues of the tooth initiated from several sites, in contrast to the mouse where the hard tissues are deposited at the same time across the whole tooth. This situation, however, appears more similar to the opossum, which shows staggered development of cusps (Jernvall and Thesleff, 2012). Most mammals also show the formation of lingual-buccal cusps in addition to the linear cusps of reptiles. This is associated with the ability to chew afforded by the development of the TMJ (temporomandibular joint) in mammals (Kermack, 1972), with Triassic Cynodonts showing similar cusp patterns to tricuspid reptiles. The folding of the epithelium to create multicuspid teeth in mammals is directed by the SEK; we therefore need to now look for evidence of such structures in multicuspid reptiles. In mammals, SEKs are identified by low proliferation, high apoptosis, and the expression of signaling factors such as *Fgf4* (Jernvall et al., 1994). An analysis of cell mechanics and gene expression within the folding inner dental epithelium on initiation of the accessory cusps in tricuspid reptiles is therefore a key next step.

In conclusions, we find two mechanisms in reptilian teeth used to generate complex shape: asymmetrical enamel deposition and folding of the inner enamel epithelium. Interestingly, both ways are used to generate complex mammalian tooth shapes indicating general mechanisms for generating complexity across the vertebrates. This study also highlights the importance of comparing similar processes, with the crest formation observed in geckos not being comparable to true cusp formation observed in other reptiles or in mammals.

### Conflict of interest statement

The authors declare that the research was conducted in the absence of any commercial or financial relationships that could be construed as a potential conflict of interest.
